# Comparison of Isolated Calcaneal Spur Excision and Plantar Fasciotomy in Addition to Spur Excision in Patients With Plantar Heel Pain Accompanied by Calcaneal Spur

**DOI:** 10.7759/cureus.31768

**Published:** 2022-11-21

**Authors:** Murat Saylik, Fırat Fidan, Osman Lapçin

**Affiliations:** 1 Orthopaedics and Trauma, Istanbul Istinye University, Istanbul, TUR; 2 Orthopaedics and Traumatology, Istanbul Aydin University, Istanbul, TUR

**Keywords:** plantar fasciitis, partial plantar fasciotomy, calcaneal spur excision, plantar fasciitis (pf), calcaneal spur, plantar heel pain

## Abstract

Introduction: The aim of this study is to clinically compare isolated calcaneal spur excision and plantar fascia release in addition to spur excision in patients with plantar heel pain accompanied by a calcaneal spur.

Method: Patients who did not benefit from conservative treatment and underwent surgical excision of the calcaneal spur and/or plantar fasciotomy were retrospectively evaluated. The patients were divided into two groups according to the surgical procedure performed. The evaluation was done according to the pre- and postoperative foot function index (FFI) using pain and functional evaluation. Pain, disability, and activity restriction were evaluated with FFI. The radiological results and FFI scores of both groups were measured before and after surgery, and the difference between the groups was compared.

Results: Of the 46 patients in our study group, 30 (65.2%) were female, and 16 (34.8%) were male. The average age was 41.2 years. There was a significant improvement in postoperative FFI scores in both groups. There was no significant difference in postoperative functional results when the groups were compared.

Conclusion: In patients whose plantar heel pain associated with calcaneal spur does not improve despite conservative treatments, both isolated spur excision and plantar fascia release in addition to spur excision may be effective treatment modalities that improve foot functions.

## Introduction

Plantar heel pain is a common clinical condition in orthopedic practice and is observed in about 10% of the population [1]. The pain is usually around the medial tubercle at the site of attachment of the plantar fascia to the calcaneus [2]. Although the exact cause of this pain is unknown, plantar fasciitis, calcaneal spur, calcaneal periostitis, and entrapment of the lateral plantar nerve are some known etiological factors [3].

Plantar calcaneal spur (PCS) is defined as the growth of calcaneus tuberosity and is observed in about 15% of the population. Although the relationship between PCS and plantar heel pain has been described before, it can also be asymptomatic [2]. It has been reported that the pain caused by PCS may be related to the size of the spur, whether it compresses the inferior calcaneal nerve or creates a micro tear in the plantar fascia, and existing inflammation [2-5].

The first treatment options for plantar heel pain accompanied by PCS are always conservative treatments [1,3,4]. Calcaneal drilling and endoscopic or open spur excisions have been described in stubborn pains that do not respond to conservative treatment [3-7]. Considering that PCS can also cause plantar fasciitis, some authors have also suggested plantar fasciotomy.

In this study, we aimed to evaluate the clinical outcomes of patients who did or did not undergo plantar fasciotomy in addition to calcaneal spur excision in patients who did not respond to conservative treatment for symptomatic PCS.

This article was previously posted to the Research Square preprint server on July 19, 2022.

## Materials and methods

Patients who applied with the complaint of heel pain between 2015 and 2021, were diagnosed with calcaneal spur clinically and radiologically, did not benefit from nonsteroidal anti-inflammatory drug use and physical rehabilitation for 12 months, and underwent surgical calcaneal spur excision and/or plantar fasciotomy were evaluated in the study retrospectively. Patients whose calcaneal spur was less than 3 mm on lateral radiography, who had a follow-up of less than six months, and who had significant pes planus, pes cavus, or other plantar arch deformity were excluded from the study. The remaining 46 patients were included in the study. The patients were divided into two groups according to the surgical procedure performed. The first group was determined as those who had undergone isolated calcaneal spur excision, and the second group was determined as patients who had undergone plantar fasciotomy in addition to calcaneal spur excision.

All patients had been treated by the same surgeon. A horizontal incision of 3-4 cm was made on the medial heel, centering the PCS through the baseline. In patients who underwent partial plantar fasciotomy (PPF), using dissection scissors not exceeding 1/2 thickness of the fascia, the proximal-central part of the fascia was loosened before PCS excision was performed, and the lateral and deep fascia parts were preserved. PCS was osteotomized under microscopic guidance. The PCS was held with forceps and peeled off from the surrounding soft tissue connections. The PCS residue was corrected with a rasp. In both groups, the length of the Achilles tendon was elongated and its tension was reduced by applying a splint that kept the ankle in 90 degrees dorsiflexion for two weeks postoperatively. After the 15th day, the splint was applied only at night and continued for a month. In both groups, the patients were mobilized without any weight-bearing for the first 15 days after the surgery, and with partial weight-bearing after the 15th day. In the first month, they were mobilized on foot, without support.

Clinical evaluation

The evaluation was done according to the pre- and postoperative foot function index (FFI) using pain and functional evaluation. Pain, disability, and activity restriction were evaluated with FFI. With the nine-parameter pain scale, the level of foot pain in various situations in daily use is measured. With the nine-parameter disability scale, the difficulty level of functional activities related to foot problems is measured. With the five-parameter activity restriction scale, the degree of activity restriction due to foot problems is measured [8]. High scores indicate more pain, disability, and restriction of activity [9].

**Figure 1:**
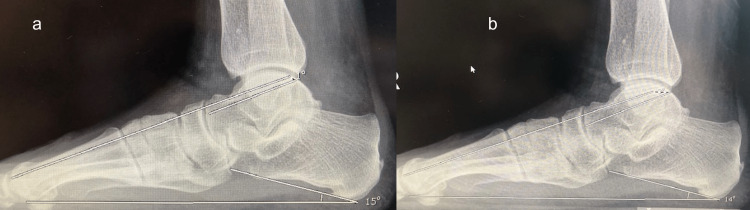
(a) Preoperative and (b) postoperative radiological images of the patient in group 1

**Figure 2:**
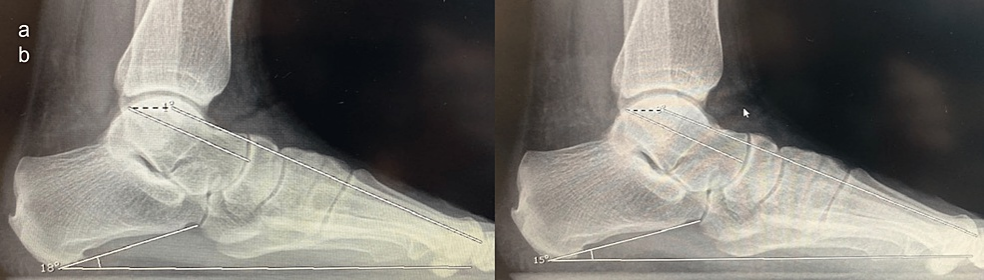
Preoperative and postoperative radiological images of the patient in group 2

The radiological results and FFI scores of both groups were measured before and after surgery and the difference between the groups was compared (Figures 1, 2). Then, the difference between the results of both groups was evaluated.

Statistical analyses of the data were done using SPSS v. 18.0 (SPSS Inc., Chicago, Illinois) in silico. Descriptive statistics were presented as minimum-maximum and mean ± standard deviation values. Preoperative and postoperative clinical scores were compared using the paired sample test. The independent sample t-test was used to compare the groups. P-value < 0.05 was considered significant.

## Results

Of the 46 patients in our study group, 30 (65.2%) were female, and 16 (34.8%) were male. The mean age was 41.2 years (range: 23-62), the follow-up period was 36.17 months (range: 14-64), and the preoperative waiting time was 16.2 months (range: 12-24) (Table 1). Of the patients, 16 underwent calcaneal spur excision. In 30 of the patients, plantar fasciotomy was performed in addition to calcaneal spur excision.

**Table 1:** General evaluation FFI: foot function index.

	Data	Mean	Median	Min	Max	Standard deviation
Age	46/46	41.2	41.5	23.0	62.0	9.81
Follow-up period (month)	46/46	36.17	35.5	14.0	64.0	14.52
Preoperative waiting time	46/46	10.17	8.5	10.0	24.0	5.47
Preoperative calcaneal pitch angle	46/46	22.15	22	18.0	26.0	6.9
Postoperative calcaneal pitch angle	46/46	18.20	18.0	14.0	24.0	6.49
Preoperative talus - 1^st^ metatarsus angle (Meary’s angle)	46/46	-1.58	-2.0	-3.0	4.0	2.49
Postoperative talus - 1^st^ metatarsus angle (Meary’s angle)	46/46	-1.82	-3.0	-4.0	3.0	2.66
Preoperative pain FFI	46/46	58.96	58.0	48.0	68.0	4.33
Postoperative pain FFI	46/46	11.15	11.0	6.0	18.0	3.05
Preoperative disability FFI	46/46	53.02	52.0	44.0	64.0	3.92
Postoperative disability FFI	46/46	9.09	8.5	4.0	15.0	2.72
Preoperative activity restriction FFI	46/46	13.17	13.5	9.0	18.0	2.54
Postoperative activity restriction FFI	46/46	4.11	4.0	2.0	10.0	1.82

The mean preoperative FFI pain score was 58.96 (range: 48-68), the mean postoperative FFI pain score was 11.15 (range: 6-18), the mean preoperative FFI disability score was 53.02 (range: 44-64), the mean postoperative FFI disability score was 9.09 (range: 4-15), the mean preoperative FFI activity restriction score was 13.17 (range: 9-18), and the mean postoperative FFI activity restriction score was 4.11 (range: 2-10).

The average PCS size was measured as 4.43 mm (min: 3; max: 8). A general evaluation table of the data was made (Table 1).

In the group where isolated PCS excision was performed, the mean preoperative FFI pain score was 58.69 (range: 54-64), and the mean postoperative FFI pain score was 12 (range: 7-18). The FFI pain score was significantly decreased (p < 0.001). The mean preoperative FFI disability score was 52.5 (range: 48-58), and the mean postoperative FFI disability score was 9.06 (range: 5-14). The FFI disability score was significantly decreased (p < 0.001). The mean preoperative FFI activity restriction score was 12.31 (range: 9-18), and the mean postoperative FFI activity restriction score was 3.88 (range: 2-9). The FFI restriction score was significantly decreased (p < 0.001).

In the group where PCS excision and PPF are applied, the mean preoperative FFI pain score was 59.1 (range: 48-68), and the mean postoperative FFI pain score was 10.7 (range: 6-16). The FFI pain score was significantly decreased (p < 0.001). The mean preoperative FFI disability score was 53.3 (range: 44-64), and the mean postoperative FFI disability score was 9.1 (range: 4-15). The FFI disability score was significantly decreased (p < 0.001). The mean preoperative FFI activity restriction score was 13.63 (range: 10-18), and the mean postoperative FFI activity restriction score was 4.23 (range: 2-10). The FFI restriction score was significantly decreased (p < 0.001).

There was no significant difference in postoperative functional results when the groups were compared (Table 2).

**Table 2:** Postoperative comparison of functional scores FFI: foot function index.

	Group 1 (n = 16)	Group 2 (n = 30)	p
FFI pain score	12 ± 3.2	10.7 ± 2.8	0.171
FFI activity restriction score	3.8 ± 1.9	4.2 ± 1.7	0.530
FFI disability score	9.6 ± 2.6	9.1 ± 2.7	0.965

## Discussion

The first step in the treatment of plantar fasciitis is conservative treatment [10]. Conservative treatment consists of physical therapy methods that will relieve tension in the plantar fascia, orthoses for the plantar region, and anti-inflammatory treatments. Of the patients, 85-90% respond to conservative treatments. In patients who do not respond to conservative treatment within 12 months, an indication for surgical treatment may occur [11].

In this study, surgical treatment was applied to patients who had plantar heel pain for 12 months, were diagnosed with plantar fasciitis, and did not respond to conservative treatments for at least 12 months. As measured by the FFI scores of patients who underwent isolated calcaneal spur excision and calcaneal spur excision combined with plantar fascia release, clinically significant improvement was observed in the foot and ankle.

Surgical treatment options for plantar fasciitis include surgical methods such as conventional open fasciotomy, endoscopic fasciotomy, neurolysis or denervation, osteotomy of the calcaneus, spur excision, or drilling [8,12-17]. As these techniques can be applied alone, combined treatment options are also applied. It has been reported that these methods have made significant improvements in functional scores in patients who have not benefited from conservative treatment [12-17]. Comparing the group in which a special rehabilitation program that strengthens the sole and corticosteroid injection was applied and the group in which PPF + PCS was applied, the FFI score at 12 months and the visual analog scale score at 24 months were found to be significantly superior and in favor of the surgical group [18]. In a study investigating patients whose pain persisted despite both conservative treatment and PPF, PCS excision was performed and pain, functional outcomes, and complications were evaluated. After PCS excision, satisfactory functional results and relief of pain were reported according to the Roles and Maudsley scoring, and PCS excision was reported as a safe surgery with a low complication rate [19]. There is no consensus on which method is superior in surgical treatment [12-18]. In several studies, it has been reported that endoscopic methods provide faster recovery and a faster return to work compared to open surgery [18-21]. In a retrospective study, where the effects of open and endoscopic surgeries were compared, it has been reported that functional results are better in the early period after endoscopic surgery, but similar functional results are observed in long-term follow-ups [22].

The etiology of plantar heel pain is complex and multifactorial [1,4]. The most common pathogenesis is considered to be plantar fasciitis [4,5]. The relationship between the calcaneal spur and plantar fasciitis is controversial [2,4,20-25]. In a recent study, the calcaneal spur was evaluated in a separate category according to the extent of the spur, and both types were reported to be associated with plantar fasciitis [4]. In another study, it was reported that the length of the calcaneal spur causes pain in plantar fasciitis and affects functional scores [26]. There is no clear indication of spur resection in the literature [21-26]. Nevertheless, some studies argue that the removal of the spur provides an improvement in pain and functional scores [3-27]. In this study, there was no significant difference in pain and clinical scores between the group that underwent plantar fascia release in addition to calcaneal spur excision and the group that underwent only spur excision.

There are some limitations of this study. The first is the small number of patients. The fact that it is single-centered and affects the genetic and psychological characteristics of the patients creates a limitation. Limitations arising from the retrospective nature of the study are among others.

## Conclusions

In conclusion, in patients with plantar heel pain who do not benefit from conservative treatment, spur excision can be performed in isolation, or additional plantar fascia release can be applied. In both surgical procedures, functionally satisfactory results can be obtained with low complication rates.
